# Time trends in management of HIV-positive pregnant women in Northern Tanzania: A registry-based study

**DOI:** 10.1371/journal.pone.0184362

**Published:** 2017-09-28

**Authors:** Tormod Rebnord, Truls Østbye, Blandina Theophil Mmbaga, Bariki Mchome, Rolv Terje Lie, Anne Kjersti Daltveit

**Affiliations:** 1 Department of Global Public Health and Primary Care (IGS), Faculty of Medicine and Dentistry, University of Bergen, Bergen, Norway; 2 Duke University, Durham, North Carolina, United States of America; 3 Kilimanjaro Christian Medical Centre, Kilimanjaro Christian Medical University College, Moshi, Tanzania; 4 Kilimanjaro Clinical Research Institute, Moshi, Tanzania; 5 Norwegian Institute of Public Health, Bergen, Norway; Universita degli Studi di Roma Tor Vergata, ITALY

## Abstract

**Objective:**

To examine time trends in antenatal factors and delivery characteristics in Northern Tanzania, and relate these to national guidelines for HIV in pregnancy.

**Design:**

Registry-based study.

**Setting:**

Northern Tanzania, 2000–2014.

**Population or sample:**

Deliveries (n = 33 346).

**Methods:**

HIV-positive women were compared with HIV-negative women during four periods spanning changing national guidelines.

**Main outcome measures:**

Known maternal HIV status, HIV treatment for woman, number of antenatal care (ANC) visits, routine folate/iron in pregnancy, anemia, delivery complications/interventions.

**Results:**

We observed an increase in deliveries with known maternal HIV status and women receiving HIV treatment, and a decline in deliveries with positive maternal HIV status (p-values for trend <0.001). The proportion of women with less than four ANC visits increased to above 30 percent irrespective of HIV status. Use of routine folate/iron increased, corresponding to a decrease in anemia which was strongest in HIV-negative women. Incidence of elective caesarean section (CS) and emergency CS remained unchanged for HIV-positive women (7.1% and 25.5%, respectively, in the last period). Use of invasive procedures declined in both groups of women. Mothers who were young, single, had low education, high parity or lived in the rural area more often had indicators of poor antenatal care.

**Conclusions:**

Increasing adherence to national guidelines over time was found for most selected outcomes. Still, a high occurrence of insufficient ANC, anemia and emergency CS call for efforts to explore and identify barriers that hinder optimal care.

## Introduction

Worldwide, 36.7 million people are living with HIV, and 2.1 million people became infected with HIV in 2015 [1]. With a total of 19 million people living with HIV, eastern and southern Africa are among the most affected regions, and these regions accounted in 2015 for 46% of the global total of new HIV infections [1]. Reported prevalence in Tanzania, with 6.2% for women and 3.8% for men [2], closely corresponds to overall prevalence for sub-Saharan Africa, although individual countries and regions differ much [3]. HIV is an exacerbating factor for maternal mortality, which declined from 842 per 100 000 live births in 2000 to 398 per 100 000 live births in Tanzania in 2015 [4]. In comparison, the global maternal mortality ratio was 216 per 100 000 live births in 2015.

Mother-to-child transmission (MTCT) accounts for over 90% of new HIV infections in young children [5]. Without preventive measures, the risk of MTCT is 15–40% in the developing world [5], and can occur during pregnancy, delivery or breastfeeding. In Tanzania overall, the MTCT rate declined from 24% in 2009 to 8% in 2015 [6], and was 9.6% in the Kilimanjaro region between 2009 and 2012 [7]. The “Tanzania National Guidelines for the Management of HIV and AIDS”, based on WHO recommendations, have been developed to improve maternal health and prevent MTCT [5]. Briefly, they target primary prevention of HIV infection among women of reproductive age, prevention of unintended pregnancies among women with HIV, prevention of transmission, as well as treatment, care and support for families living with HIV. They specifically identify risk factors for MTCT during pregnancy and delivery: premature rupture of fetal membranes (PROM), vitamin deficiency, various behavioural factors, a variety of infections, complicated deliveries and invasive delivery procedures. Caesarean section (CS) is only indicated for obstetric reasons, and not indicated in order to prevent MTCT, as long as effective antiretroviral therapy (ART) is offered. However, in circumstances where the maternal viral load is high, CS performed before the onset of labour or membrane rupture (elective CS) has been associated with reduced risk of MTCT [8–10].

The Tanzanian Prevention of Mother-to-Child Transmission (PMTCT) guidelines were introduced in 2004 and modified in 2007 and 2012 according to changes in WHO guidelines [8–14], representing changes in treatment, testing and monitoring regimens. However, adherence to and effects of implementation of the guidelines over time have not been well explored.

Therefore, the aim of this study was to examine time trends in antenatal factors and delivery characteristics identified in the national PMTCT guidelines. In particular, we wanted to investigate trends in relation to changes in these guidelines. Indicators of interest included antenatal visits, vitamin and iron supplementation, referral status, anemia and clinical management of the delivery.

## Methods

### Setting and location

The source population consists of women who delivered at Kilimanjaro Christian Medical Centre (KCMC), located in the Kilimanjaro region in Northern Tanzania. The region consists of seven districts, and has a total population of more than 1.6 million people [15]. The two main ethnic tribes in the region are Chagga and Pare, but tribes from other regions are also represented [16]. One quarter (24.2%) of the Kilimanjaro population lives in urban areas [15].

Primary health facilities in Tanzania include dispensaries providing maternal and child health care, and health centres providing inpatient care at a higher level. These health facilities have over the years integrated more comprehensive maternal and newborn services, and are often termed Reproductive and Child Health (RCH) services. Some RCH clinics may also refer patients to a Care and Treatment Clinic (CTC) for higher level of care and monitoring, especially during the postpartum period. Antenatal care (ANC), including counselling and HIV testing, is among the services offered as part of RCH services over the country. Preventive services, including ANC and services for HIV/AIDS patients, are free for all Tanzanians [17, 18].

KCMC, a referral and teaching hospital in Moshi Urban District and one of four zonal hospitals in Tanzania [19], has been one of the institutions adopting and implementing the PMTCT guidelines. It is a non-profit private hospital, based on cost-sharing, in addition to other sources of income. Although inpatient services, such as admittance and surgical procedures, involve cost-sharing fees, the antenatal services offered at KCMC are free of charge. ART for PMTCT has been free of charge since September 2004.

The Department of Obstetrics and Gynecology at KCMC includes separate units for delivery, obstetrics and gynecology. The department runs two outpatient clinics weekly, and RCH clinics the rest of the week. In addition to serving the local community in Moshi and nearby districts in the Kilimanjaro region, the department receives high risk patients from other regions.

### The birth registry and recruitment

Data was extracted from the electronic Birth Registry at KCMC, which has been in operation since July 2000 [20]. Around 3500–4000 births are registered annually, and approximately 20% of these births are cases referred from other health facilities, predominantly for medical reasons. The registry includes extensive data on mother's social background, living conditions, diseases and reproductive history. There is also detailed information on the ANC, clinical management of the delivery and the condition of both the newborn and the mother. The information in the Birth Registry is based on interviews with the mothers and on medical records. Prior to the interview, the mother is informed about the form and its contents, the intention of gathering information, and assurance of confidentiality. She is also asked to provide informed, oral consent, but can still choose not to reply to single questions. Interviews are done by trained midwives, normally within 24 hours after delivery, using a structured questionnaire.

### Data selection

We extracted data relating to a total of 49 957 births from the Birth Registry, delivered between July 2000 and July 2014. We included births with a birth weight between 500 and 7000 grams, thus excluding 187 births. For multiple deliveries we only included information on one birth, thus excluding 1332 births. Finally, to reduce selection bias and to describe a more population-based population, we included only deliveries with mother living in Moshi (Moshi urban and Moshi rural), which constitutes a natural catchment area for those giving birth at KCMC. The final analysis sample was 33 346 deliveries (S1 Fig).

### Study design and definition of terms

#### Main exposure

The main exposure in this registry-based study was HIV infection in mother during pregnancy. Maternal HIV status was stratified into three categories: HIV-negative, HIV-positive and unknown HIV status. Missing maternal HIV status was defined as unknown.

#### Time periods

Results were stratified into four time periods based on changes in national PMTCT guidelines [8–14]: 2000–2003 corresponded to the pilot phase before the national PMTCT guidelines were implemented [14], with pilot Nevirapine as the drug of choice to prevent MTCT [21].

2004–2006 corresponded to when the national guidelines were introduced and implemented, adapting WHO 2004 guidelines [13]. Two different treatment regimens were used for PMTCT, either Nevirapine or short course AZT. KCMC used the Nevirapine prophylaxis regimen, taken as a single dose tablet at the onset of labour, positive maternal HIV status being the indication. Staging of HIV/AIDS, quantitative viral load and CD4 count measurements are only referred to as required in some cases, and not as routine monitoring during ANC.

The guidelines were revised for the first time in 2007, corresponding to the third time period from 2007–2011 [8]. In this period, triple ART was the standard treatment regimen for all women with either an HIV infection classified as WHO stage 4, WHO stage 3 and CD4 <350, or CD4 <200 regardless of WHO stage. ART should continue for the rest of their lives if needed for their own health. Also, ART should be monitored during pregnancy, although the frequency of these visits is not specified.

In 2012–2014, the WHO option A was implemented, corresponding to the fourth time period [9]. All women with an HIV infection classified as WHO stage 3 or 4, or CD4 count ≤350, should receive ART, and ART should be continued for the rest of their lives if needed for their own health. The guidelines specifically speak of monitoring women with HIV every month.

The WHO Option B+ was implemented in 2014 [10, 12], and is not covered by our data. Additionally, provider-initiated testing was part of the PMTCT guidelines since they were introduced in 2004, thus from the second time period.

#### Outcome variables

The ten outcome measures obtained from current guidelines representing antenatal factors and delivery characteristics included insufficient number of antenatal care visits, not received routine folate during antenatal follow-up, not received routine iron during antenatal follow-up, referred for delivery, anemia, PROM, delivery complications, elective CS, emergency CS, and invasive procedures. Insufficient number of antenatal care visits was defined as less than four visits during the antenatal care follow-up period (ANC <4). The term “referred for delivery” implies that the woman has been referred from other health facility or by qualified health personnel to KCMC for medical reasons (risk cases), while women not referred for delivery come to KCMC on their own accord. Maternal anemia available from 2004 was defined as hemoglobin <11.0 g/dL. Values below 4 or above 19 g/dL were considered unreliable and treated as missing. The term “delivery complications” included PROM, bleeding >500 ml, third to fourth degree tear, abruption of placenta, placenta previa and other complications not specified. CS was divided into elective caesarean section and emergency caesarean section, since elective, but not emergency CS, has been associated with reduced risk of MTCT. Invasive procedures included episiotomy (89.5% of the deliveries with an invasive procedure), vacuum extraction (11.9%), forceps (0.2%) and artificial rupture of membranes (0.4%). Some births included more than one invasive procedure.

#### Other variables

Maternal age was divided into three categories (<25, 25–34, 35–49), marital status into two categories (single mother and living with partner), parity into three categories (nulliparous, second/third delivery, high parity ≥4), current residence into two categories (rural and urban, where semiurban was included in the urban category), and education level into two categories (≤ primary school and ≥ secondary school). Our data did not include detailed information on the type of treatment given to the women, and hence only a dichotomous variable indicating whether the woman received any HIV treatment during pregnancy or not, was used.

### Data analysis

Data were analysed using Stata/IC Version 14.2 for Mac. Characteristics of the study population by HIV status and time period were computed with frequency tables or with means and corresponding standard deviations. Frequency tables were further used to compare outcome measures for HIV-positive and HIV-negative women, and to assess trends in these measures over time. Adjusted relative risks and corresponding 95% confidence intervals for the dichotomous outcome measures were estimated in each time period using log binomial regression comparing HIV-positive women with HIV-negative women, presented in a forest plot. We also performed analyses to estimate the association of antenatal factors with levels of sociodemographic factors in the last time period, while testing for interactive effects between each of the sociodemographic factors and HIV status. Analyses were adjusted for maternal age, marital status, parity, current residence and education level.

In order to describe the population of women who were not referred, we conducted sensitivity analyses excluding women referred from Mawenzi Hospital, which is a public hospital located in Moshi Urban District [22], and excluding all women referred for delivery. Regression analyses excluding untreated HIV-positive women were also conducted.

### Details of ethics approval

The study was approved by the KCMC College Research Ethics Review Committee, the National Research Ethics Committee in Tanzania (NIMR/HQ/ R.8a/Vol.IX/2083 dated 3 Dec 2015), and the National Ethics Committee in Norway.

## Results

### Trends in HIV prevalence and demographics

**Fig 1:**
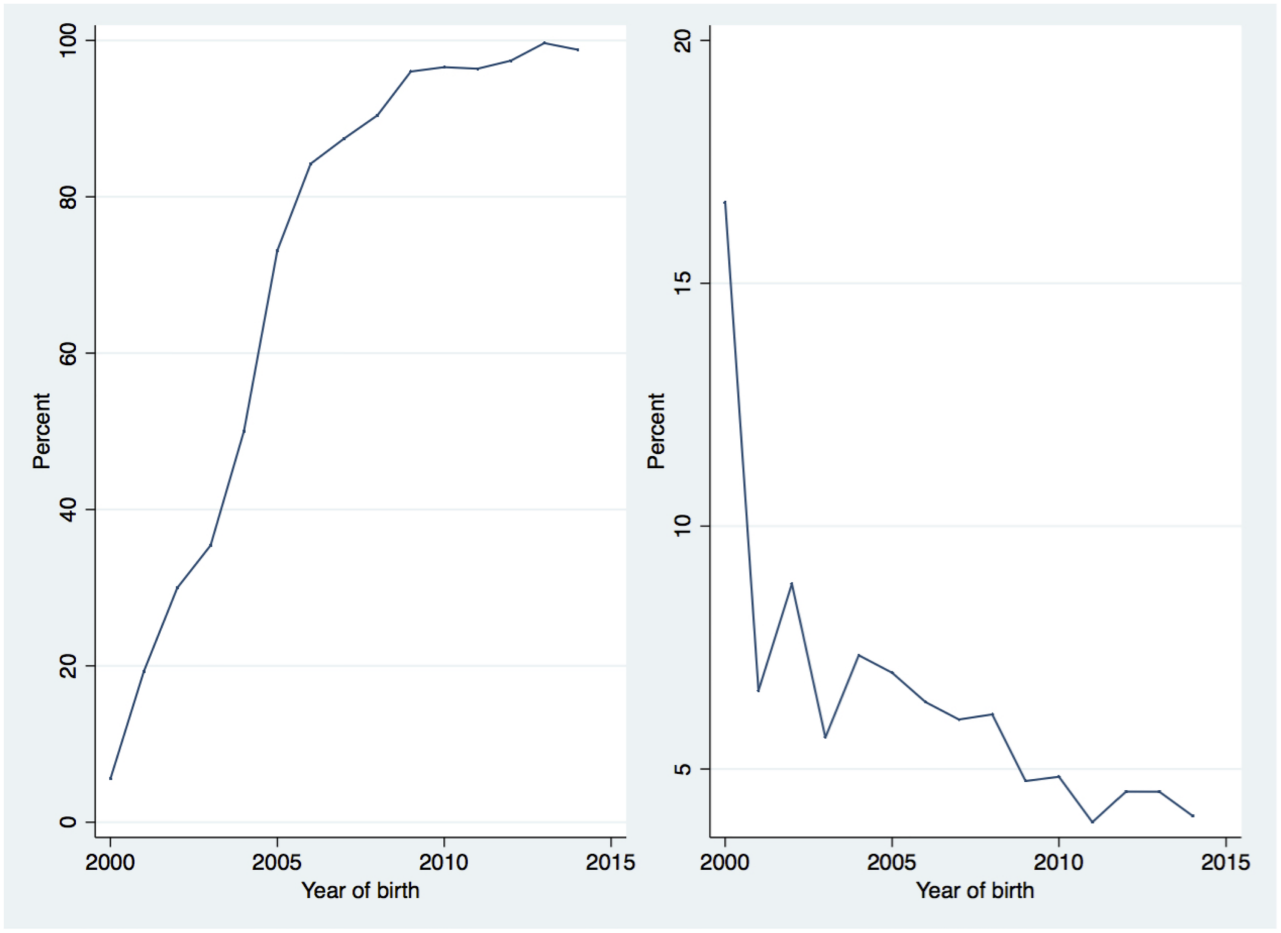
Percentage of deliveries with known maternal HIV status (left panel). Percentage of deliveries with HIV-positive mother. Denominators are deliveries with known maternal HIV status (right panel).

In total, 25 790 (77.3%) of all deliveries had a known maternal HIV status (S1 Fig), increasing from 5.4% in 2000 to 98.8% in 2014 (p-value for trend <0.001, Fig 1a). Among the women with known HIV status, the percentage who were HIV-positive declined from 17.4% in 2000 to 4.0% in 2014 (p-value for trend <0.001, Fig 1b).

Women in all categories of HIV status showed an increase in mean age from the first to the last period, HIV-positive women having the highest mean age during all periods (Table 1). In all periods HIV-positive women were less likely than HIV-negative women to live with partner or to be nulliparous, and more likely to have high parity or low education. In the first two periods HIV-positive women were also less likely to have urban residence. The percentage of women living with partner decreased through all periods for both HIV-positive and HIV-negative women. The proportion with low education decreased for all categories, but the decrease was smallest for the HIV-positive women; from 60.9% to 55.8%.

**Table 1:** Characteristics of pregnant women in Northern Tanzania by HIV status and time period.

	Time periods_1_			
	2000–03	2004–06	2007–11	2012–14
**Denominators**	*n*			
Total	6473	6055	13 700	7118
HIV-	1497	3904	12 201	6705
HIV+	115	285	642	310
HIV+ with treatment	72	232	579	281
Unknown HIV status	4861	1866	857	103
**Mother’s age**	*Mean (SD)*			
HIV-	27.2 (5.5)	27.4 (5.8)	27.6 (5.8)	28.0 (5.9)
HIV+	28.0 (5.0)	28.4 (5.5)	29.6 (5.7)	29.4 (5.8)
Unknown HIV status	26.7 (6.0)	27.0 (6.2)	27.7 (6.1)	28.4 (6.2)
**Body weight before pregn**._2_				
HIV-	64.2 (12.6)	63.4 (12.4)	63.4 (13.1)	65.0 (14.0)
HIV+	63.4 (12.2)	64.0 (14.7)	63.7 (12.4)	65.2 (12.9)
Unknown HIV status	61.3 (12.4)	62.9 (12.8)	63.3 (12.6)	64.7 (14.8)
**Living with partner**	*%*			
HIV-	92.2	90.3	87.8	85.6
HIV+	91.3	86.3	81.6	76.1
Unknown HIV status	88.2	86.0	85.3	91.2
**Nulliparous**				
HIV-	39.8	40.7	42.6	42.2
HIV+	27.0	31.2	32.4	26.5
Unknown HIV status	37.3	39.1	40.7	35.0
**High parity (≥4)**				
HIV-	13.7	14.2	12.4	12.3
HIV+	18.3	17.5	18.9	14.8
Unknown HIV status	18.8	18.2	16.6	16.5
**Urban residence**				
HIV-	72.9	75.8	74.3	71.9
HIV+	64.4	69.8	74.0	71.0
Unknown HIV status	57.7	61.7	67.6	68.0
**Education ≤ primary**				
HIV-	57.0	62.1	54.9	43.7
HIV+	60.9	68.1	61.9	55.8
Unknown HIV status	73.0	71.1	62.3	53.4

_1_ Time periods: 2000–2003: Pilot phase before national PMTCT guidelines; 2004–2006: WHO 2004 guidelines; 2007–2011: Revised WHO 2004 guidelines; 2012–2014: WHO Option A guidelines.

_2_ 6153 (18.5%) observations missing.

### Trends in management

**Fig 2:**
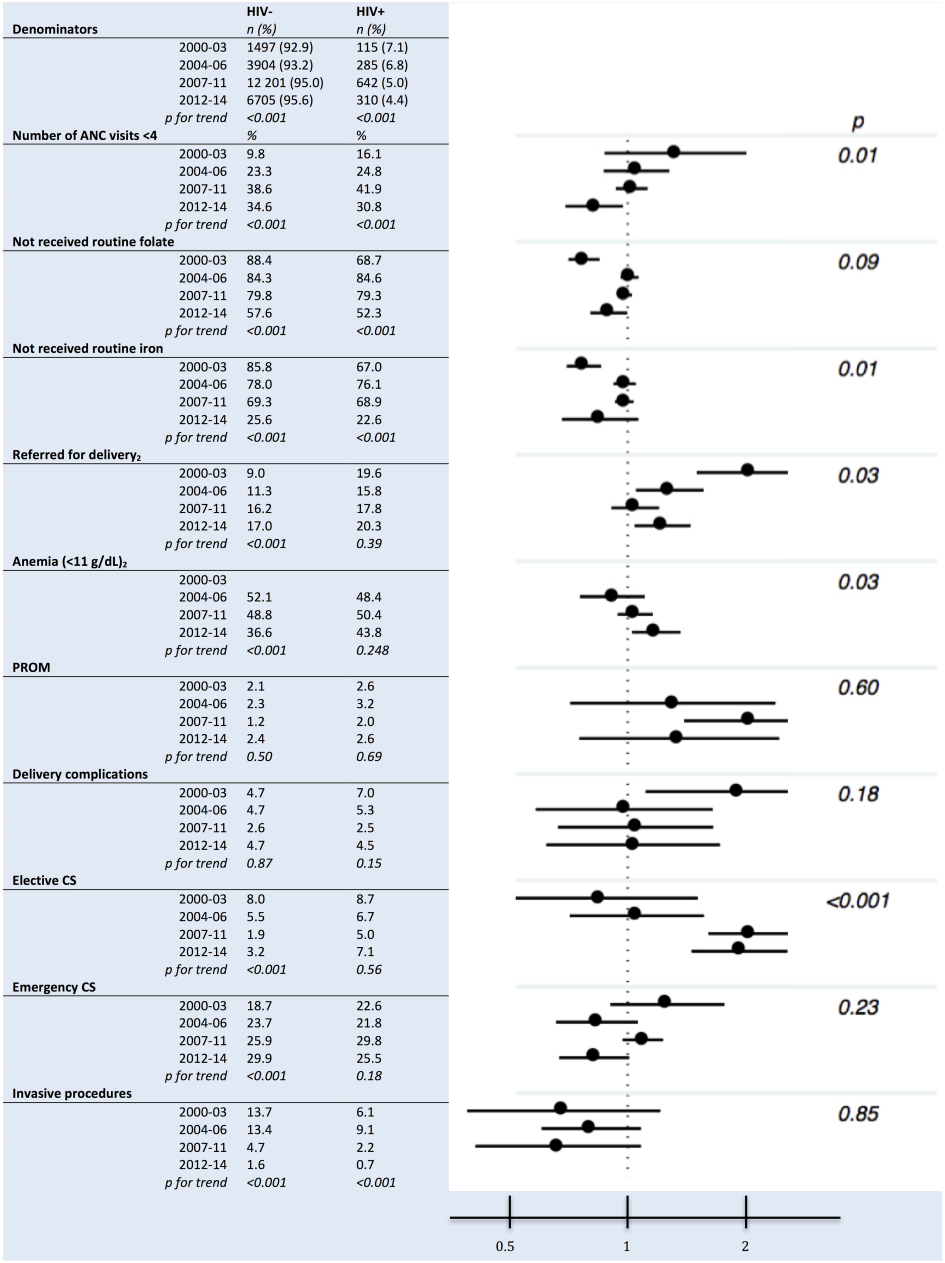
Antenatal factors and delivery characteristics among HIV-positive and HIV-negative pregnant women in Northern Tanzania by time period_1_. Forest plot shows relative risks in each time period, HIV-positive women compared with HIV-negative women. (Adjusted for maternal age, marital status, parity, current residence and education level.) P-values in plot are for trends in ARR. _1_ Time periods: 2000–2003: Pilot phase before national PMTCT guidelines; 2004–2006: WHO 2004 guidelines; 2007–2011: Revised WHO 2004 guidelines; 2012–2014: WHO Option A guidelines. _2_ Missing observations: Referred for delivery (3.8%), anemia (38.7%). Other variables <2% missing observations.

Both for HIV-positive and HIV-negative women the proportion of women with insufficient number of ANC visits increased over time (Fig 2). From the third to the last period there was a large decrease in the proportion of women who had not received routine iron and folate, and a corresponding decrease in anemia. The proportion of HIV-negative women being referred for delivery increased to nearly the same level as for HIV-positive women, which was stable through all periods. For HIV-negative women the incidence of elective CS decreased, while the incidence of emergency CS increased. The use of invasive procedures declined for both HIV-positive and HIV-negative women, the use being very low in the last period in both groups.

A reduced relative risk for HIV-positive women compared to HIV-negative women was observed over time for insufficient number of antenatal care visits and referred for delivery (p-value for trend <0.05, Fig 2). An increased relative risk over time was observed for anemia and elective CS, where adjusted relative risk increased from 0.9 to 1.2 and from 0.8 to 1.9, respectively (p-values for trend <0.05).

Among deliveries with positive maternal HIV status, 86.4% received ART. The percentage of women receiving treatment was 63.2% in the first time period, 82.3% in the second, 90.3% in the third, and 90.7% in the last time period (p-value for trend <0.001).

### Sociodemographic differences

Women with less than the recommended number of ANC visits were more likely to be young (<25), to have high parity, to be a single mother, or to have low education (Table 2). Women who did not receive folate and iron were more likely to live in the urban area. Women who were referred for delivery were more likely to be young, to be nulliparous, to have low education, or to live in the rural area. Women with anemia were more likely to be young, or to have low education. Still, we found no statistically significant interactions with HIV status.

**Table 2:** Percentage and adjusted relative risk (ARR) of selected indicators of pregnancy care by maternal sociodemographic characteristics. Northern Tanzania (time period 4).

	ANC visits <4		No folate/iron		Referred for delivery		Anemia	
Sociodemographic factors	%	ARR (95% CI)	%	ARR (95% CI)	%	ARR (95% CI)	%	ARR (95% CI)
**Maternal age (n)**								
<25 (2165)	36.4	1.32 (1.22–1.43)	19.7	1.04 (0.92–1.17)	27.8	1.67 (1.52–1.83)	42.2	1.27 (1.18–1.38)
25–34 (3806)	31.7	ref	20.9	ref	12.2	ref	34.2	ref
35–49 (1129)	39.0	0.98 (0.90–1.08)	22.2	1.03 (0.91–1.18)	13.9	1.03 (0.90–1.19)	37.0	1.06 (0.96–1.18
**Parity (n)**								
Nulliparous (2946)	28.8	0.71 (0.65–0.77)	18.6	0.79 (0.71–0.89)	22.0	1.58 (1.43–1.74)	36.7	0.96 (0.88–1.04)
Second/third (3282)	35.5	ref	22.5	ref	12.3	ref	37.1	ref
≥4 (890)	48.7	1.40 (1.28–1.54)	21.7	0.97 (0.84–1.13)	19.0	1.26 (1.10–1.45)	37.2	0.99 (0.89–1.12)
**Marital status (n)**								
Single mother (1050)	36.6	1.26 (1.15–1.38)	20.6	1.07 (0.93–1.23)	23.4	1.04 (0.96–1.14)	36.3	0.95 (0.86–1.05)
Living with partner (6058)	34.0	ref	20.7	ref	16.1	ref	37.0	ref
**Education (n)**								
≤ primary (3153)	39.5	1.16 (1.09–1.24)	20.5	0.97 (0.88–1.07)	24.7	1.84 (1.70–2.01)	39.8	1.13 (1.05–1.21)
≥ secondary (3959)	30.3	ref	20.9	ref	11.1	ref	34.9	ref
**Residence (n)**								
Rural (2007)	37.9	1.07 (1.00–1.14)	17.7	0.81 (0.72–0.91)	35.7	2.98 (2.71–3.26)	37.6	0.99 (0.92–1.06)
Urban (5111)	33.0	ref	21.9	ref	9.9	ref	36.7	ref

Missing observations: Referred for delivery (3.8%), anemia (38.7%). Other variables <2% missing observations. Adjusted for maternal age, marital status, parity, current residence and education level.

In the most recent time period, being untreated was associated with rural residence (14.4% untreated), while only 7.3% of women with urban residence were untreated (p-value 0.049).

### Sensitivity analyses

Excluding women referred from Mawenzi Hospital or any woman referred for delivery resulted in lower rates of elective and emergency CS both for HIV-positive and HIV-negative women in most of the time periods. However, changes in time trends and relative risks were modest both for CS and the other factors (data not shown). Neither did analyses excluding untreated women change the results substantially.

## Discussion

In this registry-based study of 33 346 deliveries in Northern Tanzania, we saw an increase in the proportion of deliveries with known maternal HIV status and the proportion of HIV-positive women who received treatment, and a decrease in the proportion of deliveries with positive maternal HIV status. Other time trends that correspond with guidelines for HIV in pregnancy were a decrease in the proportion of women not receiving folate and iron, a decrease in the proportion with anemia, and a decrease in use of invasive procedures. Time trends not corresponding with guidelines were an increase in the proportion of insufficient number of ANC visits, and a stronger decline in anemia among HIV-negative women than HIV-positive women. There were also clear trends in CS. While in the first period there were small differences between HIV-positive and HIV-negative women, HIV-positive women were more likely to have an elective CS and HIV-negative women more likely to have an emergency CS in the last period.

### Trends in HIV prevalence and demographics

Our data showed that women who were tested for HIV in 2000 were more likely to be HIV-positive than women who were tested in 2014. This may not represent a true decline in HIV prevalence among pregnant women in this region, as those selected for HIV test in the early years most likely were at higher risk of infection. However, our results correspond with a decrease in overall HIV prevalence in Tanzania [23, 24]. Increased counselling and interventions by the national health authorities [5, 25, 26] may have contributed to a real decline by preventing new cases of HIV in the population. Also, increased use of contraception among HIV-positive women might have reduced the number of unintended pregnancies [27].

### Antenatal management of pregnant HIV-positive women

The large increase in proportion of deliveries with known maternal HIV status signifies a considerable change in awareness and knowledge about the HIV threat.

#### Number of antenatal care visits

A less favorable trend was seen for number of ANC visits for both HIV-positive and HIV-negative women. Despite a decline from the third to the last time period, the proportion of women with less than the four recommended ANC visits was higher in the last period than in the first two periods. We also found that young maternal age, low education, single motherhood, high parity and living in the rural area were associated with low attendance to ANC. Both the decline in number of women with recommended number of ANC visits and socio-demographic associates with ANC attendance are in line with national statistics from Tanzania [28–30]. Gupta et al. has reported that higher quality of services, HIV testing and counselling, and higher educational status of the woman were among the factors positively associated with ANC utilization, while never married woman and long distance to health facility were factors negatively associated with ANC utilization [31]. Our results, together with these findings, emphasize the need for high-quality, easy accessible ANC services, with an urgent need to reach vulnerable women characterized by these factors. ANC services, including medication and vitamin supplementation, are all free of charge, and the number of ANC visits or lack of treatment and supplementation should therefore not be influenced by this factor. However, there is reason to believe that the payment exemptions might vary depending on what medical facility the patient is visiting (private, government-run or faith-based) [18]. Also, the cost of transport to the ANC clinics may be an economic factor influencing the number of visits.

#### Referral

While most women who deliver at KCMC come on their own accord, some women are referred to KCMC from other hospitals or health facilities due to high risk. The proportion being referred for delivery was stable around 16–20 percent for HIV-positive women (Fig 2). Referral might indicate suboptimal planning of delivery, or a delay in receiving appropriate care in time. Planning of mode and place for delivery in good time is part of the national guidelines for HIV in pregnancy [5]. Although the referred women should benefit from the higher level of care given at KCMC, delivery for HIV-positive women might comply better with the guidelines for preventing PMTCT, if the women were first admitted to KCMC in a timely fashion. Services at public hospitals and health facilities are, in contrast to certain services at KCMC, exempted from cost-sharing fees. This might attract women who should have sought KCMC in the first place.

#### Multivitamin supplementation

Daily folic acid and iron supplementation in pregnant women increased both among HIV-positive and HIV-negative from the third to the last period (Fig 2). Supplementation is part of Tanzanian national guidelines as a key component of antenatal care for pregnant women [5], and is also included in global WHO recommendations to improve pregnancy outcomes and to reduce maternal anemia [32].

#### Changes in anemia prevalence

Parallel to the increased use of folic acid and iron, we observed a decreased prevalence of anemia. Although we cannot confirm a causal relationship based on our data, it is reasonable to assume that increased use of folic acid and iron in accordance with national guidelines has reduced maternal anemia over time. The largest decline in anemia was, however, seen in HIV-negative women. We did not have information on duration of HIV treatment/prophylaxis, but we speculate whether the use of zidovudine (AZT) might explain the slower decline in anemia for HIV-positive women. Anemia is a common side effect of zidovudine (AZT), which was part of the treatment regimen at KCMC in both the third and the fourth time period [8, 9, 13]. Nevertheless, option B+, a WHO option which was rolled out in Tanzania during 2014 and therefore not covered by our data, offers more alternatives to the use of zidovudine (AZT) [10]. Hopefully, this will reduce the occurrence of anemia among HIV-positive pregnant women.

### Management of delivery of HIV-positive women

Over time, the incidence of both elective and emergency CS remained unchanged for HIV-positive women (7.1% and 25.5%, respectively, in the last period), while for HIV-negative women use of elective CS decreased and use of emergency CS increased (3.2% and 29.9%, respectively, in the last period, Fig 2). These trends are reflected in an increased relative risk of elective CS for HIV-positive women compared with HIV-negative women (Fig 2).

In our data, the emergency CS rate was close to 30% during 2000–2014 and the elective CS rate was slightly above 3%. Sørbye et al. presented CS rates at KCMC in the period 2000–07 according to the Robson Ten-Group Classification [33]. They reported high CS rates even in the low-risk groups, and especially for referred women; the total CS rate was 55.0% among referred women and 26.9% among women who came on their own accord. In comparison, reported CS rate has in our study period varied between 3 to 6% in Tanzania overall, and between 7.2 and 11.0% in the Kilimanjaro region [28–30].

Although a CS is not indicated for the purpose of reducing MTCT, national guidelines and studies [5, 8–10, 34] report that elective CS has been associated with a reduced risk of MTCT in HIV-positive women who did not receive ART. Because few HIV-positive women were untreated after 2007, we did not assess trends in the elective CS rate among untreated HIV-positive women. However, the different distribution of elective and emergency CS according to HIV status might indicate that a careful risk assessment of HIV-positive women is being done. These results should therefore encourage to continued efforts to obtain optimal use of elective CS in HIV-positive women, although the main focus should be to identify how to reduce the proportion of emergency CS for all women.

The cost of delivery and CS at KCMC has gradually increased [35]. The association between referral and high CS rate, combined with the high rate of emergency CS, may reflect that cost-sharing fees are a barrier for women to seek appropriate care in the first place. We consider HIV-positive pregnant women a vulnerable group that to a greater extent could benefit from payment exemption, also in inpatient situations, such as deliveries.

### Who are the women who don’t receive optimal pregnancy care?

Young maternal age, low education, single motherhood, high parity and living in the rural area were associated with one or more indicators of poor antenatal care (Table 2). Delivery care in this area is based on referral of women from other health facilities to KCMC in situations where a risk for complicated delivery is identified. But the fact that young women, women with low education, and single mothers are all more likely to be referred, should be a concern. Living in the rural area, and having high parity, are also probable barriers to antenatal care. These results might indicate lack of time, opportunity, proximity and economy as possible deterrents to proper care. More in-depth interviews with these women would lead to a better understanding of these barriers.

### Strengths and limitations

A major strength is that data in the birth registry have been systematically collected by structured interviews on a daily basis over the whole time span. The study is based on a large number of observations, allowing us to assess time trends, although not for the least common outcomes. Also, exclusion of women living outside the local area of Moshi may have reduced any potential selection bias.

Still, differences between those who give birth at KCMC and the general pregnant population in the area could affect our results. Potential factors influencing place of delivery are economy, residence and knowledge, and a known barrier to deliver at KCMC is that admittance and surgical procedures are based on cost-sharing fees, in contrast to public hospitals where fees are usually absent or minimal for pregnant women. This has been discussed in several paragraphs. However, there are exemptions from payment at KCMC for patients who cannot afford to pay these fees. There is also a considerable amount of home deliveries in the Kilimanjaro region (11.9% in 2010) [29].

Information on anemia was missing among 38.7% of the women. The possibility that HIV-positive women are more likely to be checked for anemia, represents a risk of differential misclassification bias that could lead to higher risk estimates among HIV-positive women compared with HIV-negative women. Numbers for PROM are likely underestimated, as other studies report higher proportions with PROM [36, 37].

We were not able to assess results related to type of ART, as only information about treatment given or not given is available in the registry. It is, however, reasonable to believe that treatment given was in accordance with guidelines although there may have been local variations in when new guidelines were adapted.

## Conclusion

Increasing adherence to national guidelines over time was found for many of the recommendations. Still, a considerable proportion of HIV-positive women are untreated, have an insufficient number of ANC visits, have anemia, and emergency CS is still common. Young maternal age, single motherhood, low education, high parity and living in the rural area were associated with poor antenatal care, and we suggest further studies to explore and to identify barriers in order to give proper antenatal care and achieve better adherence to national guidelines.

## Supporting information

S1 Fig Selection and description of study population. (TIFF)

## References

[1] Fact Sheet 2016 [Internet]. Joint United Nations Programme on HIV/AIDS (UNAIDS); 2016 [cited 2016 12 June]. http://www.unaids.org/sites/default/files/media_asset/20150901_FactSheet_2015_en.pdf.

[2] Tanzania Commission for AIDS (TACAIDS) ZACZ, National Bureau of Statistics (NBS), Office of the Chief Government Statistician (OCGS), ICF International. Tanzania HIV/AIDS and Malaria Indicator Survey 2011–12: Key Findings [Internet]. Dar es Salaam, Tanzania2013 [cited 2016 12 June]. http://www.dhsprogram.com/pubs/pdf/SR196/SR196.pdf.

[3] The Gap Report 2014 [Internet]. 2014 [updated Sept 2014; cited 2016 June 12]. http://www.unaids.org/sites/default/files/media_asset/UNAIDS_Gap_report_en.pdf.

[4] Maternal mortality ratio (modeled estimate, per 100,000 live births) [Internet]. The World Bank. [cited June 08 2017]. http://data.worldbank.org/indicator/SH.STA.MMRT?name_desc=false.

[5] Tanzania National Guidelines for the Management of HIV and AIDS [Internet]. Tanzania Ministry of Health and Social Welfare; 2012 [cited 2016 12 June]. 4th:[http://pmtct.or.tz/wp-content/uploads/2013/03/ART-guidelines_PDF.pdf.

[6] United Republic of Tanzania [Internet]. Joint United Nations Programme on HIV/AIDS (UNAIDS); 2016 [cited 2016]. Fact sheet]. http://www.unaids.org/sites/default/files/media/documents/UNAIDS_GlobalplanCountryfactsheet_tanzania_en.pdf.

[7] Mwendo EM, Mtuy TB, Renju J, Rutherford GW, Nondi J, Sichalwe AW, et al Effectiveness of prevention of mother-to-child HIV transmission programmes in Kilimanjaro region, northern Tanzania. Trop Med Int Health. 2014;19(3):267–74. Epub 2014/01/07. doi: 10.1111/tmi.12255 . 24386998 10.1111/tmi.12255

[8] Prevention of Mother-to-Child Transmission of HIV, National Guidelines [Internet]. Tanzania Ministry of Health and Social Welfare; 2007 [cited 2016 Nov 15]. http://apps.who.int/medicinedocs/documents/s19272en/s19272en.pdf.

[9] National guideline for comprehensive care of prevention of mother-to-child transmission of HIV services [Internet]. Tanzania Ministry of Health and Social Welfare; 2012 [cited 2016 June 12]. 3rd:[http://pmtct.or.tz/wp-content/uploads/2013/03/Tz-PMTCT_Guidelines_AUGUST_FINAL.pdf.

[10] National guideline for comprehensive care services for prevention of mother-to-child transmission of HIV and keeping mothers alive [Internet]. Tanzania Ministry of Health and Social Welfare; 2013 [cited 2016 June 12]. http://pmtct.or.tz/wp-content/uploads/2013/10/tz_guidelines_ccs_optionb_all.pdf.

[11] Countdown To Zero, Elimination of New HIV Infections Among Children by 2015 And Keeping Their Mothers Alive [Internet]. UNICEF; 2012 [cited 2016 June 12]. https://www.unicef.org/aids/files/hiv_pmtctfactsheetTanzania.pdf.

[12] Gamell A, Letang E, Jullu B, Mwaigomole G, Nyamtema A, Hatz C, et al Uptake of guidelines on prevention of mother-to-child transmission of HIV in rural Tanzania: time for change. Swiss Med Wkly. 2013;143:w13775 Epub 2013/03/23. doi: 10.4414/smw.2013.13775 . 23519621 10.4414/smw.2013.13775

[13] Tanzania Ministry of Health and Social Welfare. National Guidelines for Prevention of Mother-to-child Transmission of HIV. 2004.

[14] Wilfert C, Bonanno L, Haule A, Muze B, Mwangomale A, Msofe JY, et al. Foundation-supported PMTCT Program Evaluation. The Elizabeth Glaser Pediatric AIDS Foundation, 2010.

[15] Population Distribution by Age and Sex [Internet]. National Bureau of Statistics Ministry of Finance Dar es Salaam, Office of Chief Government Statistician President’s Office, Finance, Economy and Development Planning Zanzibar; 2013 [cited 2016 June 12]. http://ihi.eprints.org/2169/1/Age_Sex_Distribution.pdf.

[16] Habib NA, Daltveit AK, Bergsjo P, Shao J, Oneko O, Lie RT. Maternal HIV status and pregnancy outcomes in northeastern Tanzania: a registry-based study. Bjog. 2008;115(5):616–24. Epub 2008/03/13. doi: 10.1111/j.1471-0528.2008.01672.x . 18333943 10.1111/j.1471-0528.2008.01672.x

[17] Research NIfM. Evidence-informed policy making in the United Republic of Tanzania: setting REACH-policy initiative priorities for 2008–2010. National Institute for Medical Research, 2008 September 2008. Report No.

[18] Babbel BE. Evaluating Equity in the Provision of Primary Health Care in Tanzania. In: University OS, editor. 2012.

[19] Background Information [Internet]. Kilimanjaro Christian Medical Centre; [cited 2016 Nov 15]. http://www.kcmc.ac.tz/?q=background.

[20] Bergsjo P, Mlay J, Lie RT, Lie-Nielsen E, Shao JF. A medical birth registry at Kilimanjaro Christian Medical Centre. East Afr J Public Health. 2007;4(1):1–4. Epub 2007/10/03. . 17907753

[21] Afnan-Holmes H, Magoma M, John T, Levira F, Msemo G, Armstrong CE, et al Tanzania's countdown to 2015: an analysis of two decades of progress and gaps for reproductive, maternal, newborn, and child health, to inform priorities for post-2015. Lancet Glob Health. 2015;3(7):e396–409. Epub 2015/06/20. doi: 10.1016/S2214-109X(15)00059-5 . 26087986 10.1016/S2214-109X(15)00059-5

[22] Hospital description [Internet]. 2016 [cited 2016 June 13]. http://www.electives.net/hospital/6435/preview.

[23] Prevalence of HIV, total (% of population ages 15–49) [Internet]. The World Bank. [cited June 13 2016]. http://data.worldbank.org/indicator/SH.DYN.AIDS.ZS/countries/1W-TZ?display=graph).

[24] Global AIDS Response Country Progress Report [Internet]. Joint United Nations Programme on HIV/AIDS (UNAIDS); 2014. http://www.unaids.org/sites/default/files/country/documents/TZA_narrative_report_2014.pdf.

[25] National behaviour change communication guidelines on HIV and AIDS interventions [Internet]. Tanzania Commission for AIDS (TACAIDS); 2012 [cited 2016 Nov 15]. http://tacaids.go.tz/tacaids/images/sampledata/documents/BCC-GUIDELINES-TACAIDS-A4fnl.pdf.

[26] Consolidated guidelines on the use of antiretroviral drugs for treating and preventing HIV infection: recommendations for a public health approach [Internet]. World Health Organization; 2013 [cited 2016 Nov 15]. 2nd:[http://apps.who.int/iris/bitstream/10665/208825/1/9789241549684_eng.pdf?ua=1. 27466667

[27] Contraceptive prevalence, any methods (% of women ages 15–49) [Internet]. The World Bank. [cited Nov 15 2016]. http://data.worldbank.org/indicator/SP.DYN.CONU.ZS?end=2010&locations=TZ&start=1992&view=chart.

[28] (NBS) NBoS. Tanzania Demographic and Health Survey 2004–05. Dar es Salaam, Tanzania: 2005.

[29] (NBS) NBoS. Tanzania Demographic and Health Survey 2010. Internet. National Bureau of Statistics (NBS), ICF Macro, 2011 Contract No.: Nov 15.

[30] Ministry of Health CD, Gender, Elderly and Children (MoHCDGEC). Tanzania Demographic and Health Survey and Malaria Indicator Survey (TDHS-MIS) 2015–16. Dar es Salaam, Tanzania, and Rockville, Maryland, USA: 2016.

[31] Gupta S, Yamada G, Mpembeni R, Frumence G, Callaghan-Koru JA, Stevenson R, et al Factors associated with four or more antenatal care visits and its decline among pregnant women in Tanzania between 1999 and 2010. PLoS One. 2014;9(7):e101893 doi: 10.1371/journal.pone.0101893 . 25036291 10.1371/journal.pone.0101893 PMC4103803

[32] Guideline: Daily iron and folic acid supplementation in pregnant women [Internet]. Geneva: World Health Organization; 2012 [cited 2016 Nov 15]. http://apps.who.int/iris/bitstream/10665/77770/1/9789241501996_eng.pdf?ua=1. 23586119

[33] Sorbye IK, Vangen S, Oneko O, Sundby J, Bergsjo P. Caesarean section among referred and self-referred birthing women: a cohort study from a tertiary hospital, northeastern Tanzania. BMC Pregnancy Childbirth. 2011;11:55 Epub 2011/07/30. doi: 10.1186/1471-2393-11-55 . 21798016 10.1186/1471-2393-11-55 PMC3160415

[34] Read JS N M. Efficacy and safety of cesarean delivery for prevention of mother-to-child transmission of HIV-1. Cochrane Database of Systematic Reviews. 2005;(4). 10.1002/14651858.CD005479 PMC12861580 16235405

[35] Nilsen C, Ostbye T, Daltveit AK, Mmbaga BT, Sandoy IF. Trends in and socio-demographic factors associated with caesarean section at a Tanzanian referral hospital, 2000 to 2013. Int J Equity Health. 2014;13:87 doi: 10.1186/s12939-014-0087-1 . 25319518 10.1186/s12939-014-0087-1 PMC4206704

[36] Cho G, Min K-J, Hong H-R, Kim S, Hong J-H, Lee J-K, et al High-risk human papillomavirus infection is associated with premature rupture of membranes. BMC Pregnancy and Childbirth. 2013;13(1):1–4. doi: 10.1186/1471-2393-13-173 24011340 10.1186/1471-2393-13-173 PMC3846597

[37] Parry S, Strauss JF. Premature Rupture of the Fetal Membranes. New England Journal of Medicine. 1998;338(10):663–70. doi: 10.1056/NEJM199803053381006 . 9486996 10.1056/NEJM199803053381006

