# Correction: Time trends in management of HIV-positive pregnant women in Northern Tanzania: A registry-based study

**DOI:** 10.1371/journal.pone.0209545

**Published:** 2018-12-18

**Authors:** Tormod Rebnord, Truls Østbye, Blandina Theophil Mmbaga, Bariki Mchome, Rolv Terje Lie, Anne Kjersti Daltveit

There is an error in the Methods section under the sub-heading “Details of ethics approval.” The correct statement is: The birth registry project at Kilimanjaro Christian Medical Centre obtained ethical clearance from the Tanzania Ministry of Health, and from the Kilimanjaro Christian Medical College (KCM-College) research ethics committee. The project also applied for approval from a Norwegian IRB, which stated that they did not need to approve the project. This study obtained additional clearance certificates from National Institute for Medical Research (NIMR) in Tanzania (NIMR/HQ/R.8a/Vol. IX/2073) and from Kilimanjaro Christian Medical University College (research proposal 696, certificate number 819).
